# Supplementary figures and images for: Saccharomyces cerevisiae Mus81-Mms4 prevents accelerated senescence in telomerase-deficient cells
Source: PLoS Genet. 2020 May 29;16(5):e1008816. doi: 10.1371/journal.pgen.1008816 (PMC7286520; doi:10.1371/journal.pgen.1008816)

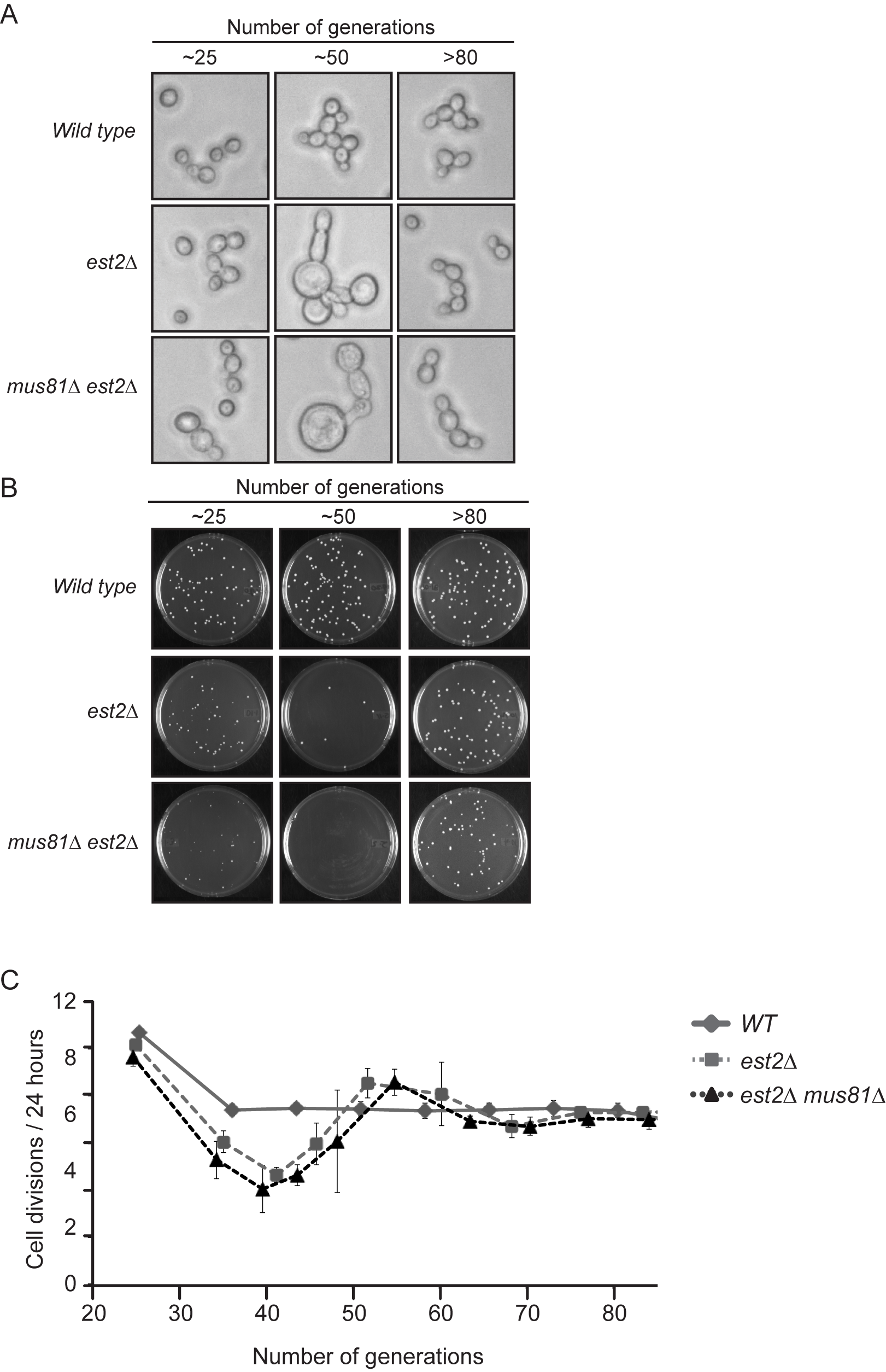

Supplement: S1 Fig — (A) Cell morphology was documented using bright-field light microscopy. Images were taken during early-senescence (~25 generations), late-senescence (~50 generations) and post-senescence (>80 generations). (B) Cell viability was monitored daily by plating 100 or 500 cells and allowing 2–5 days of growth for colony formation. Images were taken for visual documentation of colony growth at generation times 25 and 50. Post-senescence generation times were 80 or 100 for est2-deficient and wild type strains, respectively. (C) The number of cell divisions per 24-hour period are plotted over the total number of generations for wild type (WT), telomerase deficient est2Δ cells, and est2Δ mus81Δ double mutant strains. Error bars represent standard deviation of the number of cell divisions per 24-hour period for at least seven independent spore clones at each generation. All haploid strains were derived from WDHY2961 as described in Materials and Methods. (TIF) [file pgen.1008816.s001.tif]

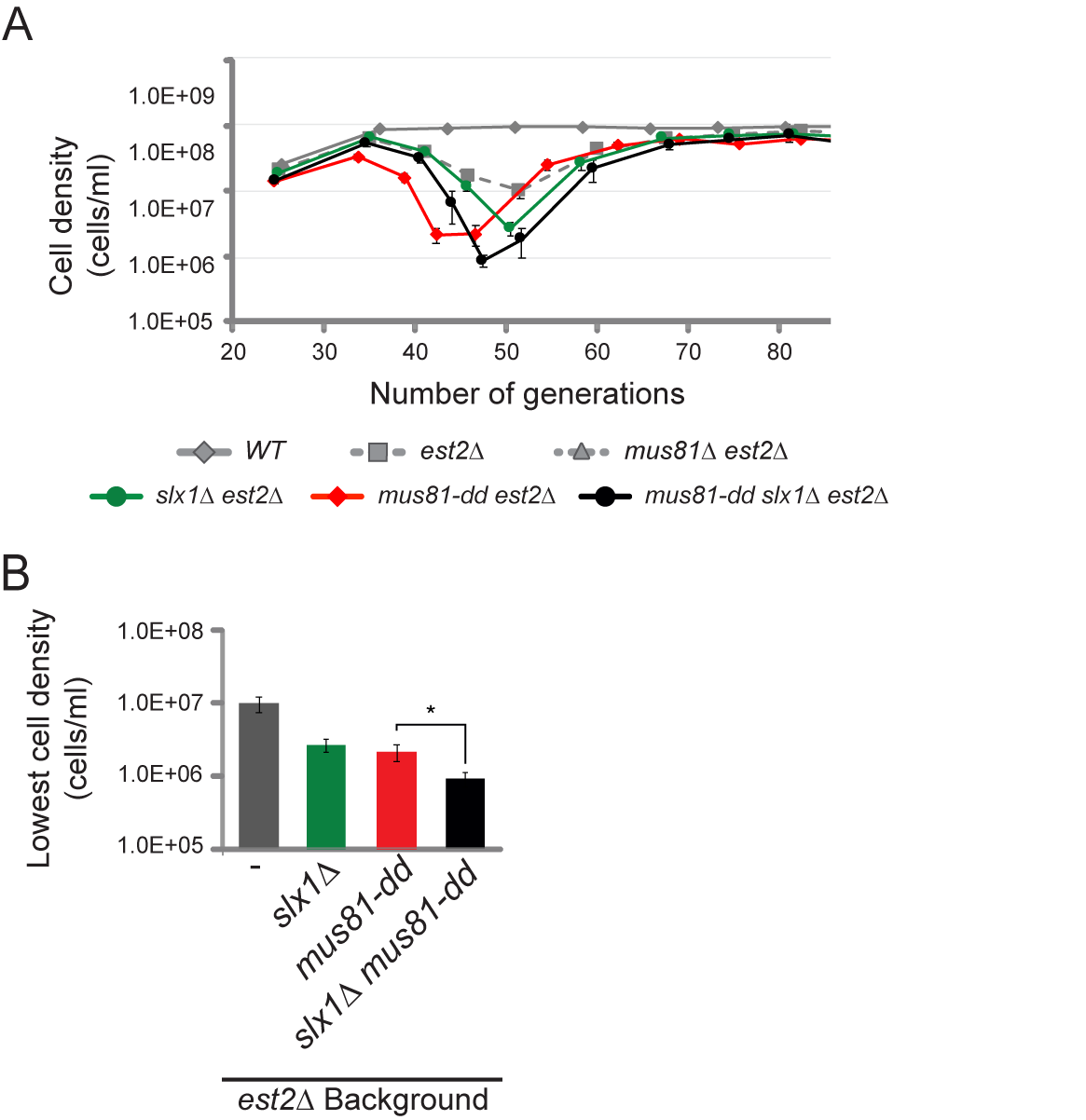

Supplement: S2 Fig — (A) Serial dilution assays monitoring cell density after 24 hours from an initial inoculate of 5x10^5^ cells. Average cell density and one standard error is plotted at the given generations for wild type (WT) (n = 20), est2Δ (n = 40), est2Δ mus81Δ (n = 27), est2Δ slx1Δ (n = 24) and est2Δ mus81-D414,415A (mus81-dd) (n = 23) and est2Δ mus81-D414,415A (mus81-dd) slx1Δ (n = 7) strains. Haploid strains in (A) were generated by sporulation of WDHY3007 (WT, est2Δ and est2Δ mus81Δ) and WDHY3114 (est2Δ slx1Δ and est2Δ mus81-D414,415A (mus81-dd)) as described in Materials and Methods. (B) Average cell density was plotted with one standard error at the generation with the lowest average cell density for strains in (A). Statistics compare mean values. Student T-test is * P < 0.05. (TIF) [file pgen.1008816.s002.tif]

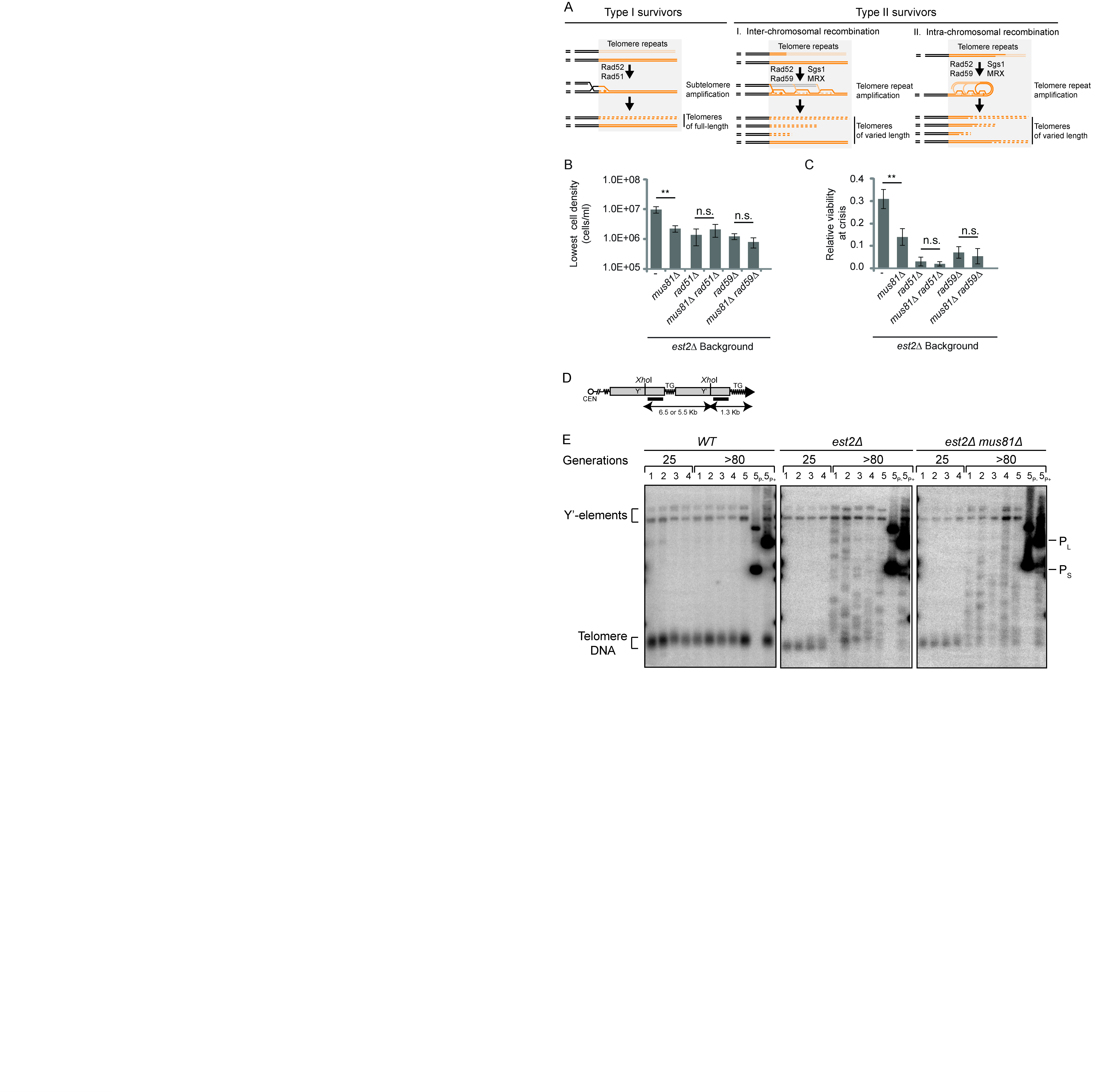

Supplement: S3 Fig — (A) Proposed mechanisms for Type I and Type II alternative-telomere lengthening and involved proteins. Differences in survivor telomere length are highlighted at the bottom of the diagram. (B) The lowest average cell density and one standard error was plotted from serial dilution experiments shown in Fig 3 for strains est2Δ (n = 40), est2Δ mus81Δ (n = 27), est2Δ rad51Δ (n = 8) and est2Δ mus81Δ rad51Δ (n = 10), est2Δ rad59Δ (n = 9) and est2Δ mus81Δ rad59Δ (n = 9). Statistics were performed to compare conditions with and without MUS81. Student T-test is * P < 0.05, ** P < 0.01, and n.s. = not significant. (C) Average relative cell viability was calculated at the generation with the lowest average cell density (senescence crisis) for the strains in (B). Haploid strains rad51Δ, mus81Δ rad51Δ, est2Δ rad51Δ, and est2Δ rad51Δ mus81Δ strains were derived from diploid WDHY3358 as described in Materials and Methods. Remaining haploid strains, est2Δ rad59Δ, and est2Δ rad59Δ mus81Δ, were derived from WDHY3366. WT and est2Δ strains were derived from sporulation of both diploids. (D) Terminal telomeres and subtelomeric Y’-elements were freed from the chromosome by XhoI digest which cuts internal to the Y’-element region. Depending on the telomere length and subtelomere composition, Southern blot analysis using the Y’ probe can monitor both telomere repeat length and the status of the Y’-elements [85]. (E) Southern analysis of DNA from early- and post-senescence cells from wild type (WT), est2Δ, and est2Δ mus81Δ genetic backgrounds collected from liquid media. Genomic DNA was probed using an oligonucleotide complementary to the Y’-element region adjacent to the telomere indicated in (D). Brackets indicate the terminal XhoI fragments and Y’-containing telomeres. Independent survivor isolates are numbered above each lane for both early- and post-senescent cell populations. Plasmid controls (P) are shown in the last two lanes to control for enzymatic digest, with and without XhoI (+ [file pgen.1008816.s003.tif]

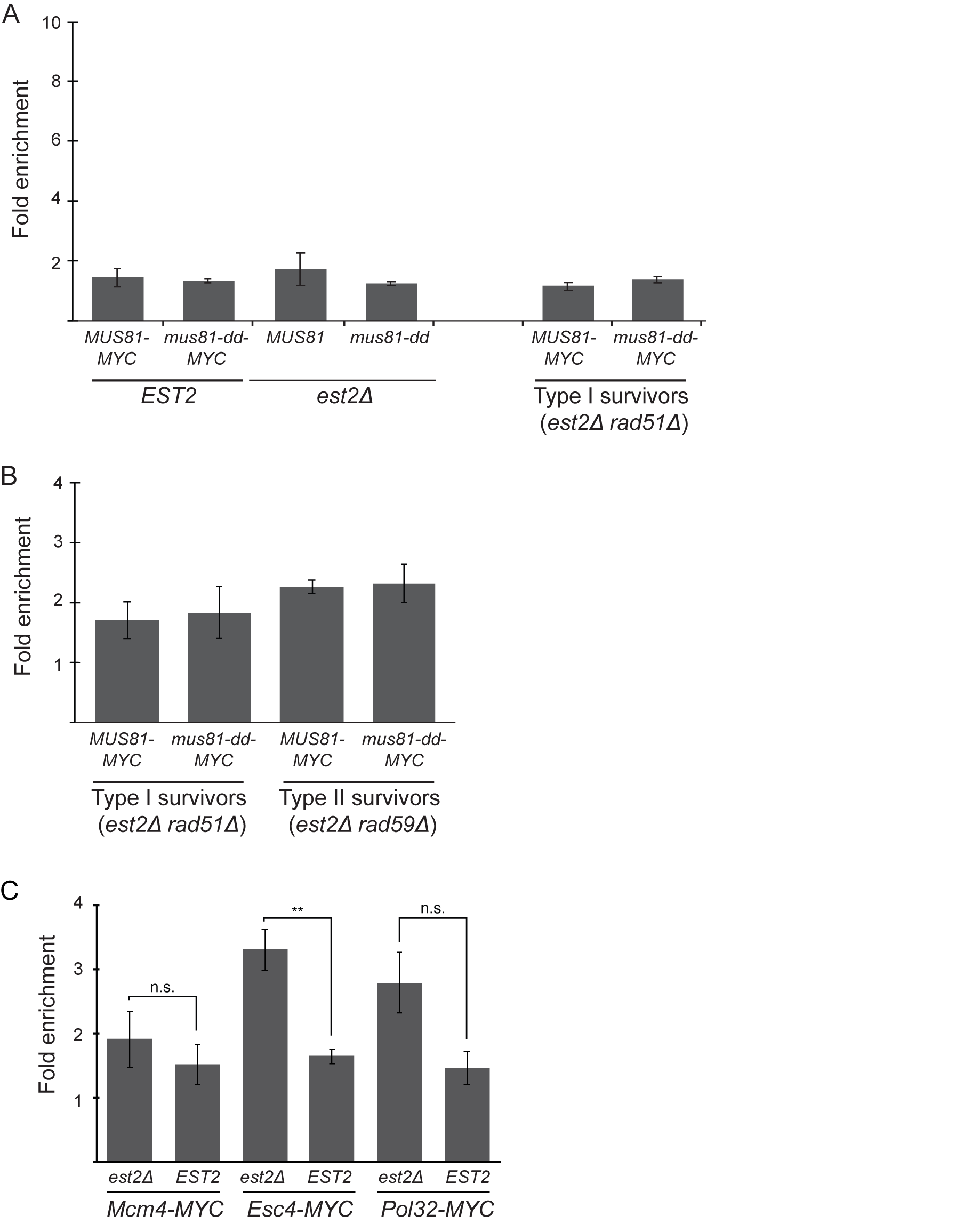

Supplement: S4 Fig — (A) Cells were collected after survival from replicative senescence and subjected to ChIP using anti-Myc antibody [87]. Serial streaks were prepared of est2Δ rad51Δ yeast cells with either MUS81-9MYC or mus81-D414,D415A-9MYC to acquire the survivor strains. Average fold-enrichment of three replicates and a single standard error are presented for each strain. Samples were normalized to input samples and fold-enrichments calculated as Y’subtelomeric DNA over SAM1 non-telomeric DNA. (B) Similar as in (A), serial streaks were prepared of est2Δ rad59Δ yeast cells with either MUS81-9MYC or mus81-D414,D415A-9MYC to acquire the survivor strains. Average fold-enrichment of three replicates and a single standard error are presented for each strain. Samples were normalized to samples without antibody and fold-enrichments calculated as Y’subtelomeric DNA over SAM1 non-telomeric DNA. (C) Association of Mcm4-Myc, Esc4-9Myc, and Pol32-9Myc was performed either in EST2 or telomerase deficient (est2Δ) strain backgrounds. Average fold enrichment of three experimental replicates are presented with a single standard of error. Student T-test is * P < 0.05, ** P < 0.01, and n.s. = not significant. Strains were derived from diploids WDHY2961, WDHY2962, and WDHY3007 for est2Δ mus81Δ, est2Δ MUS81-MYC, and est2Δ mus81-dd-MYC respectively. Similar haploids were derived from diploids WDHY5296 (est2Δ rad51Δ MUS81-9MYC and est2Δ rad51Δ mus81-D414,D415A-9MYC) and WDHY5297 (est2Δ rad59Δ MUS81-9MYC and est2Δ rad59Δ mus81-D414,D415A-9MYC). (TIF) [file pgen.1008816.s004.tif]

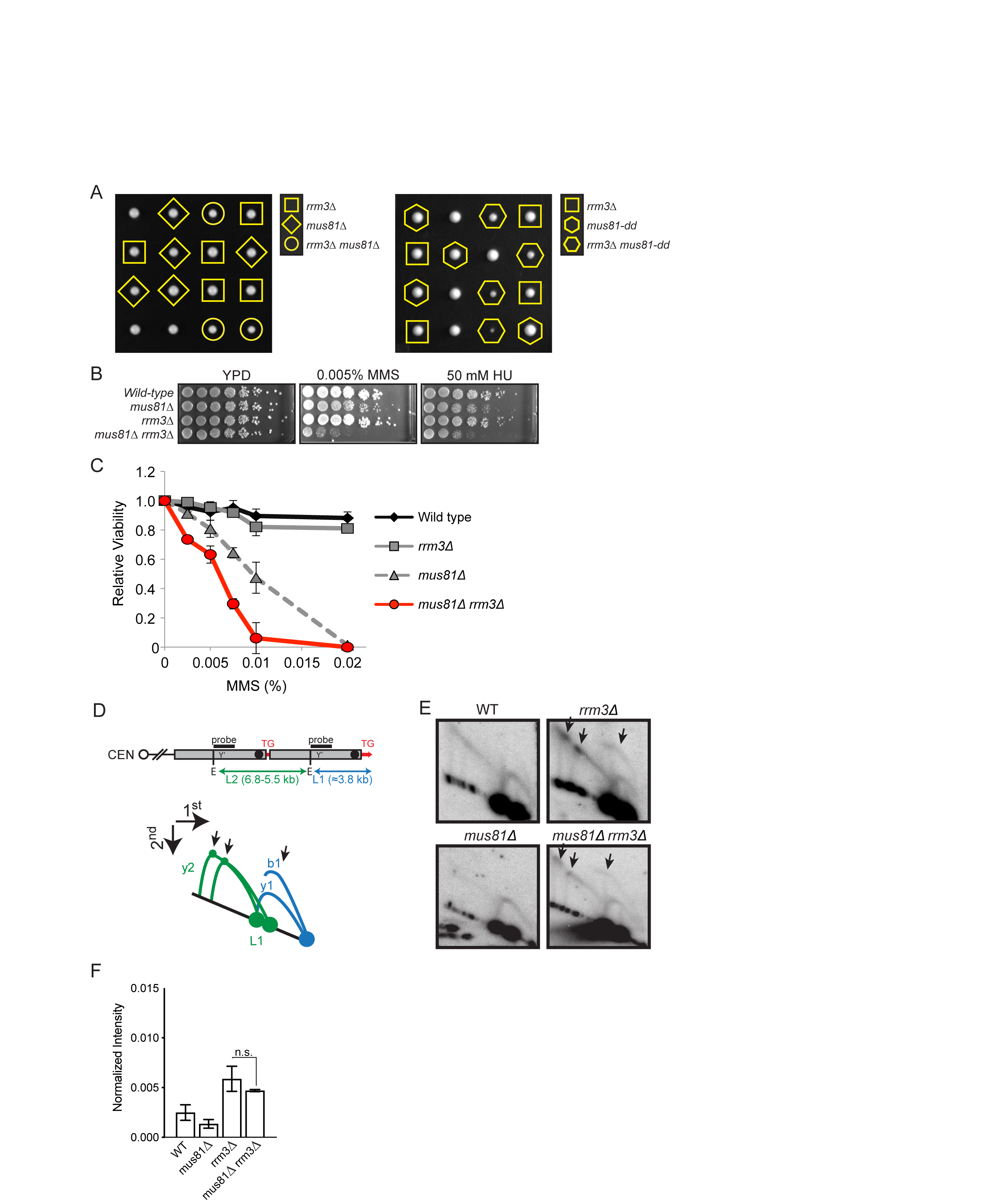

Supplement: S5 Fig — (A) Diploid cells heterozygous for mutations in MUS81 and RRM3 were created by mating WDHY3638 and WDHY2272 or WDHY2835. Sporulated haploid spores were allowed to grow on nutrient-rich media for 2–3 days. Colony size was documented and four-spore tetrads were assessed for growth markers related to rrm3::KanMX and mus81::URA3, while PCR amplification and digest identified mus81-D414,415A (mus81-dd). [Yellow squares = rrm3Δ; yellow diamond = mus81Δ; yellow circles = rrm3Δ mus81Δ; Yellow hexagon (point vertical) = mus81-dd; Yellow hexagon (point horizontal) = rrm3Δ mus81-dd] (B) Indicated strains were assessed by chronic exposure to methyl methanesulfonate (MMS) and hydroxyurea (HU), wild type (W303-RAD5 MATα), mus81Δ (WDHY1858), rrm3Δ (WDHY3638), and mus81Δ rrm3Δ (WDHY3606). (C) Viability of haploid yeast in (B) was assessed under conditions of chronic exposure to increasing concentrations of MMS or left untreated. Average relative viability was plotted at every concentration for at least 5 clones with a single standard error. (D) Schematic representation of a chromosome arm containing a tandem arrangement of two Y’ subtelomeric elements and the associated replication intermediates profile by 2D-gel. The Y’ elements contain an ARS (black circle) and an EcoRI site “E”. The presence of telomeric TG tracts, at both the telomere and between the two Y’ elements, is shown in red. Digestion with EcoRI will produce two fragments: the terminal L1 fragment (blue) and an internal L2 fragment (green) which can be 5.5 or 6.8 kb-long. Both the L1 and L2 fragments mainly migrate as Y structures (y1 and y2). Arrows indicate the expected structures upon replication fork pausing at TG tracts in the rrm3Δ mutant: a bubble structure (b1) for the terminal fragment when the distal fork is block at the telomere, and a local increase of signal along the y2 arcs upon stalling at the internal TG tract (67). (E) Representative 2D-gel analysis of sub-telomeric and telomeric replication intermedi [file pgen.1008816.s005.tif]

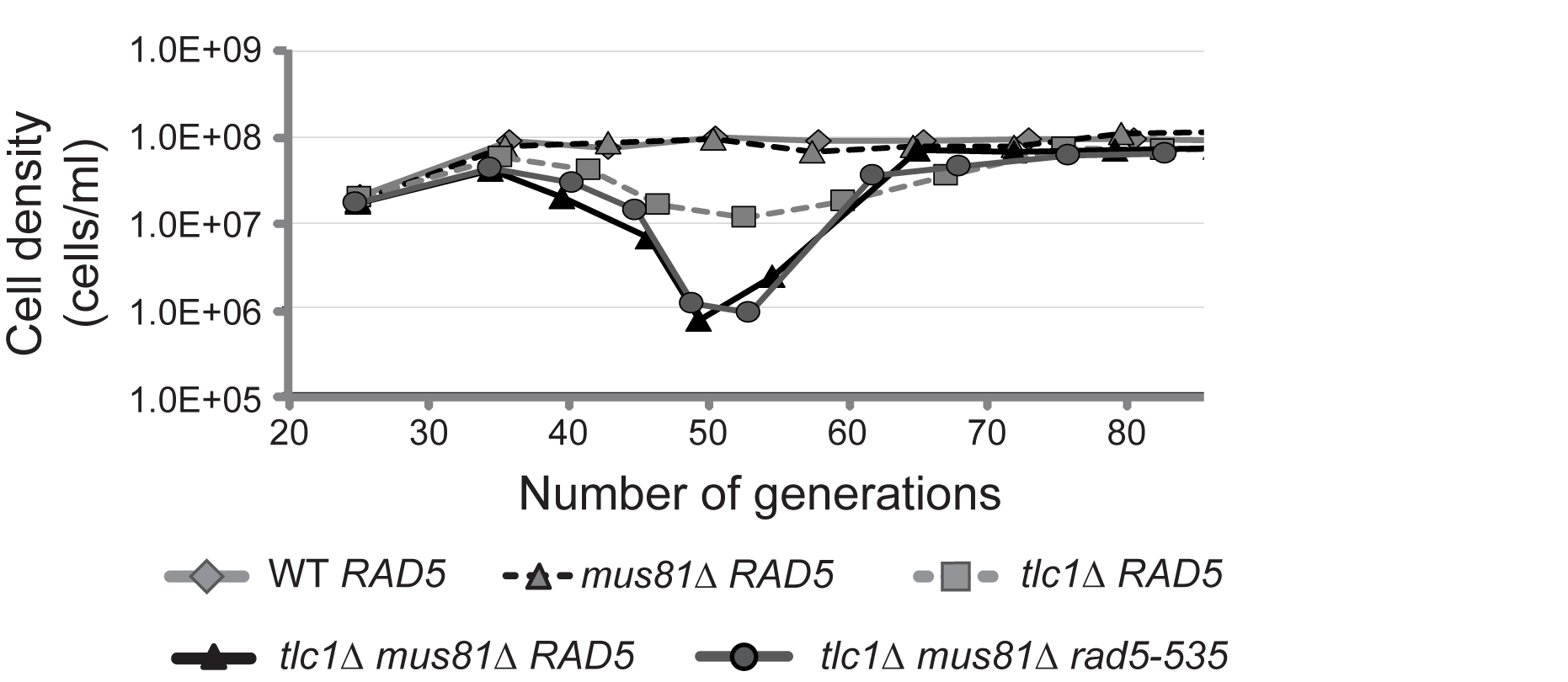

Supplement: S6 Fig — (A) Serial dilution assays monitoring cell density after 24 hours from an initial inoculate of 5x10^5^ cells. Average cell density is plotted at the given generations for wild type (WT) RAD5 (n = 4), mus81Δ RAD5 (n = 3), tlc1Δ RAD5 (n = 8), tlc1Δ mus81Δ RAD5 (n = 4), tlc1Δ mus81Δ rad5-535 (n = 4) strains. Haploid strains were generated by sporulation of WDHY3651 as described in Materials and Methods. (TIF) [file pgen.1008816.s006.tif]

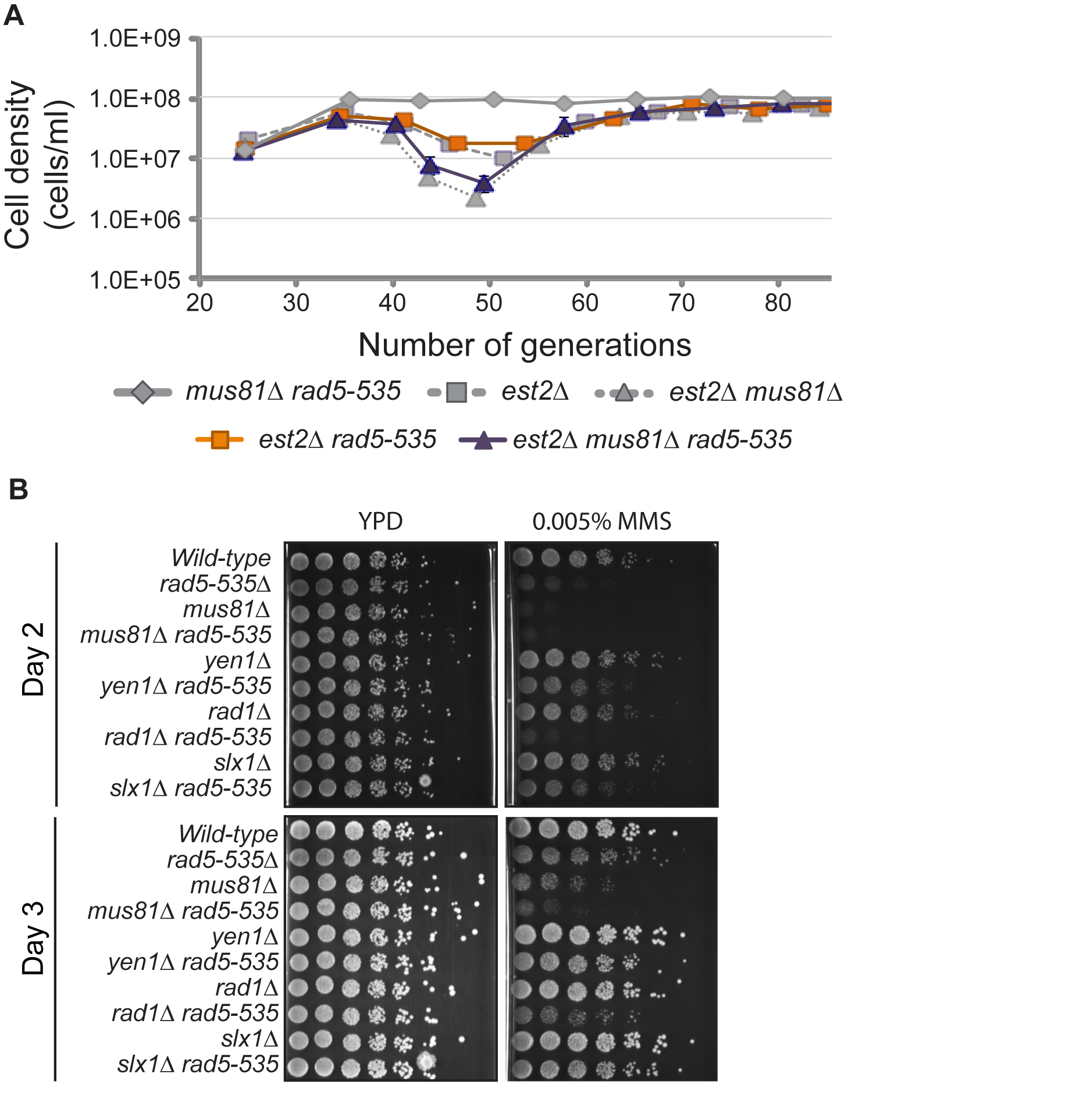

Supplement: S7 Fig — (A) Serial dilution assays monitoring cell density after 24 hours from an initial inoculate of 5x10^5^ cells. Average cell density of at least eight haploid spores is plotted at the given generations for rad5-535 mus81Δ (n = 8), est2Δ (n = 40), est2Δ mus81Δ (n = 26), est2Δ rad5-535 (n = 8) and est2Δ mus81Δ rad5-535 (n = 8) strains. Haploid strains in (A) were generated by sporulation of WDHY5327 as described in Materials and Methods. (B) Haploid yeast strains were assessed by chronic exposure to methyl methanesulfonate (MMS). Strains included wild type (W303-RAD5), rad5-535 (W303), mus81Δ (WDHY1858), mus81Δ rad5-535 (JMY380), yen1Δ (WDHY2755), yen1Δ rad5-535 (WDHY3105), rad1Δ (WDHY3106), rad1Δ rad5-535 (WDHY3161), slx1Δ (WDHY3148), and slx1Δ rad5-535 (WDHY3113). (TIF) [file pgen.1008816.s007.tif]

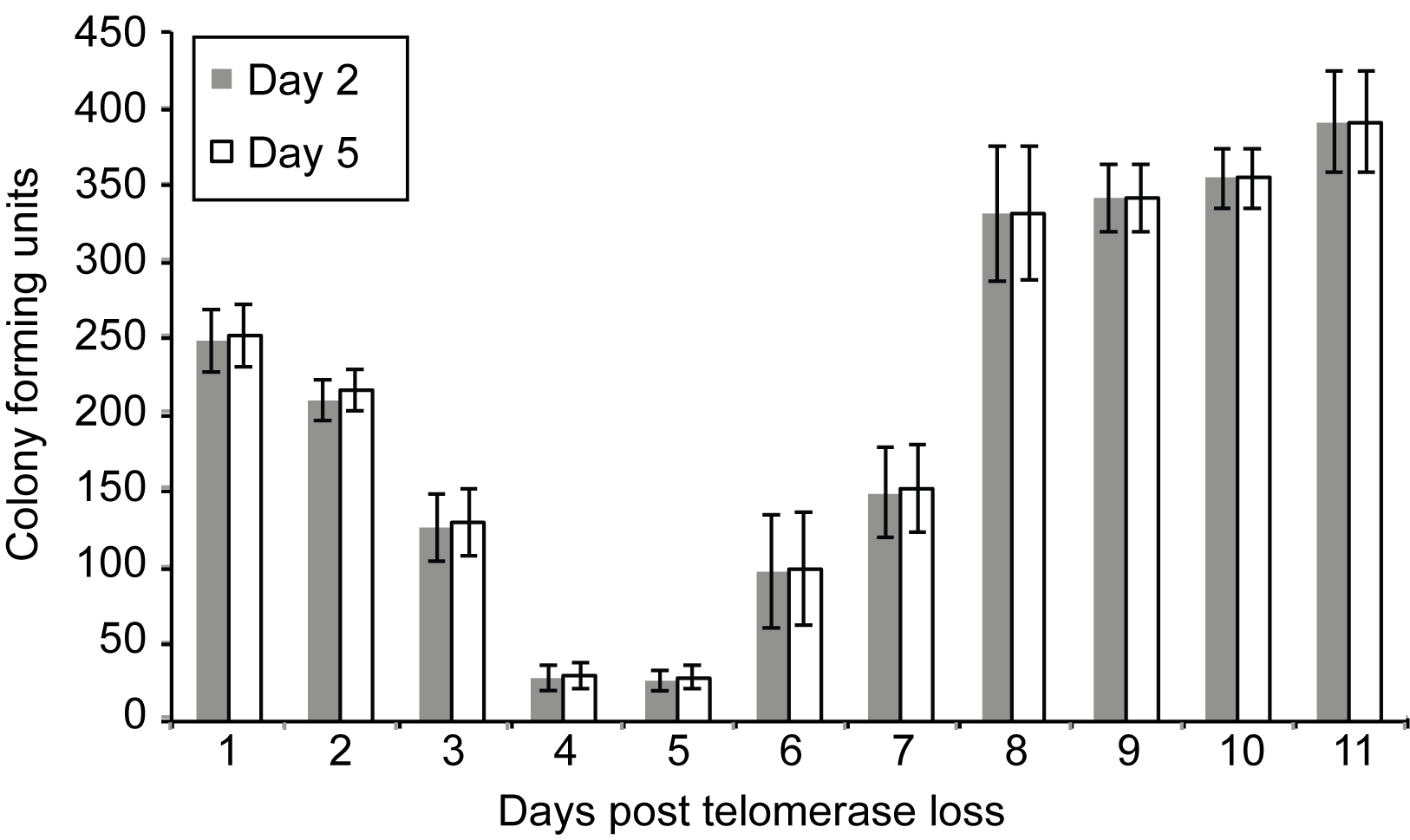

Supplement: S8 Fig — As part of the serial dilution assay, cell bodies were counted, and predetermined number of cells plated to assess viability. Visible colony forming units were counted regardless of colony size. Average numbers of colonies are presented with one standard error. Haploid strains were generated by sporulation of WDHY3007 (WT, est2Δ and est2Δ mus81Δ) as described in Materials and Methods. (TIF) [file pgen.1008816.s008.tif]
