# Supplementary material for: Saccharomyces cerevisiae Mus81-Mms4 prevents accelerated senescence in telomerase-deficient cells
Source: PLoS Genet. 2020 May 29;16(5):e1008816. doi: 10.1371/journal.pgen.1008816 (PMC7286520; doi:10.1371/journal.pgen.1008816)
Supplement: S1 Table — (DOCX) [file pgen.1008816.s009.docx]

**S1 Table. *Saccharomyces cerevisiae* strains**

| **Strain** | **Relevant genotype** | **Source** |
| --- | --- | --- |
| W303^ǂ^ | *MAT a; ade2-1 can1-100 his3-11,15 leu2-3,112 trp1-1 ura3-1 rad5-G535R* | S. Elledge |
| W303-*RAD5** | *MAT a; ade2-1 can1-100 his3-11,15 leu2-3,112 trp1-1 ura3-1 RAD5* | R. Rothstein |
| W303-*RAD5** | *MAT α; ade2-1 can1-100 his3-11,15 leu2-3,112 trp1-1 ura3-1 RAD5* | R. Rothstein |
| y816^ǂ^ | *MAT a; cdc31-1 rad5-G535R* | S. Elledge |
| 1922-1B^ | *MAT a; ADE2 can1-100 his3∆3’-311 HOcs(117) leu2-3,112 trp1-1 URA3 RAD3 rad1::LEU2* | A. Bailis |
| X1914^ | *MAT a/α; ade2-1/ade2-1 CAN1/CAN1 his3-11/HIS3 leu2-3,112/leu2-3,112 ura3-1/ura3-1 trp1-1/trp1-1 est2::ura3::LEU2/EST2 rad52::TRP1/RAD52 hxt::URA3/HXT13 RAD5/RAD5* | A. Bailis |
| W6241-2A* | *MAT a; W303 ADE2 TRP1 LYS2 rrm3::KanMX leu29∆*EcoRI*::URA3::leu2∆*BsteII | R. Rothstein |
| JMY380^ǂǂ^ | W303-*RAD5 MAT* a*; mus81::KanMX rad5-G535R* | S. Brill |
| WDHY1858 | W303-*RAD5 MAT α; mus81::KanMX* | Lab collection |
| WDHY2272 | W303-*RAD5 MAT α; mus81::URA3* | Lab collection |
| WDHY2601 | W303-*RAD5 MAT* a*; mus81::KanMX* | Lab collection |
| WDHY2602 | W303-*RAD5 MAT α; mus81::KanMX* | Lab collection |
| WDHY2755 | W303-*RAD5 MAT a; yen1::loxP-KanMX-loxP* | Lab collection |
| WDHY2835 | W303-*RAD5 MAT α; ura3::loxp mus81-D414,415A* | Lab collection |
| WDHY2961 | WDHY3006; *est2::ura3::LEU2/EST2 mus81::KanMX/MUS81 mms4::TRP1/MMS4* | This study |
| WDHY2962 | WDHY3006; *est2::ura3::LEU2/EST2 mus81-9Myc::TRP1/MUS81 bar1::KanMX/BAR1* | This study |
| WDHY3006 | *MAT a/α; ade2-1/ade2-1 can1-100/CAN1 his3-11/his3-11 leu2-3,112/leu2-3,112 ura3-1/ura3-1 trp1-1/trp1-1 est2::ura3::LEU2/EST2 RAD5/RAD5* | This study |
| WDHY3007 | WDHY3006; *est2::ura3::LEU2/EST2 mus81-D414,415A-9Myc::TRP1/MUS81 bar1::KanMX/BAR1* | This study |
| WDHY3027 | WDHY3006; *est2::ura3::LEU2/EST2 slx1::loxP-KanMX-loxP/SLX1* | This study |
| WDHY3036 | WDHY3006; *est2::ura3::LEU2/EST2 yen1::loxP-KanMX-loxP* | This study |
| WDHY3052 | W303-*RAD5 MAT α; cdc13-1 mus81-D414,415A-9Myc::TRP1* | This study |
| WDHY3054 | W303-*RAD5 MAT α; cdc13-1 rad1::LEU2* | This study |
| WDHY3056 | W303-*RAD5 MAT α; cdc13-1 yen1::loxP-KanMX-loxP* | This study |
| WDHY3058 | W303-*RAD5 MAT α; cdc13-1 mus81::KanMX* | This study |
| WDHY3083 | WDHY3006; *est2::ura3::LEU2/EST2 mus81::KanMX/MUS81 slx1::loxP-KanMX-loxP/SLX1* | This study |
| WDHY3085 | W303*-RAD5 MAT a; cdc31-1* | This study |
| WDHY3086 | W303-*RAD5 MAT α; cdc31-1* | This study |
| WDHY3112 | W303-*RAD5 MAT α; cdc13-1 slx1::loxP-KanMX-loxP* | This study |
| WDHY3105  WDHY3106  WDHY3113 | W303 *MAT α yen1::loxP-KanMX-loxP rad5-G535R*  W303 *MAT a rad1::LEU2 rad5-G535R*  W303 *MAT a; slx1::loxP-KanMX-loxP rad5-G535R* | This study  This study  This study |
| WDHY3114 | W303-*RAD5 est2::ura3::LEU2/EST2 mus81-D414,415A-9Myc::TRP1/MUS81 slx1::loxP-KanMX-loxP/SLX1* | This study |
| WDHY3143 | W303 *MAT* *α*; *mus81-dd-9Myc:TRP rad5-G535R* | This study |
| WDHY3145 | WDHY3006; *est2::ura3::LEU2/EST2 rad1::LEU2/RAD1* | This study |
| WDHY3148 | W303-*RAD5 MAT a; slx1::loxP-KanMX-loxP* | Lab collection |
| WDHY3161 | W303 *MAT* *α*; *rad1::LEU2 rad5-G535R* | This study |
| WDHY3358 | WDHY3006; *est2::ura3::LEU2/EST2 mus81::KanMX/MUS81 rad51::LEU2/RAD51* | This study |
| WDHY3366 | WDHY3006; *est2::ura3::LEU2/EST2 mus81::KanMX/MUS81 rad59::LEU2/RAD59* | This study |
| WDHY3369 | WDHY3006; *est2::ura3::LEU2/EST2 mus81::KanMX/mus81::KanMX rad51::LEU2/RAD51* | This study |
| WDHY3605 | W303-*RAD5 MAT a; mus81::KanMX rrm3::KanMX* | This study |
| WDHY3606 | W303-*RAD5 MAT α; mus81::KanMX rrm3::KanMX* | This study |
| WDHY3634 | W303-*RAD5 MATa; rrm3::KanMX mus81-D414,415A* | This study |
| WDHY3638 | W303*-RAD5 MAT a; rrm3::KanMX* | This study |
| WDHY3639 | W303*-RAD5 MAT α; rrm3::KanMX* | This study |
| WDHY3651 | *MAT a/α; ade2-1/ade2-1 can1-100/ can1-100 his3-11/his3-11 leu2-3,112/leu2-3,112 ura3-1/ura3-1 trp1-1/trp1-1 ssd1s (psi+) tlc1::LEU2/TLC1 mus81::KanMX/MUS81 rad5/RAD5* | This study |
| WDHY3653 | WDHY3006; *est2::ura3::LEU2/EST2 mus81::KanMX/MUS81 rrm3::KanMX/RRM3* | This study |
| WDHY3660 | W303*-RAD5 MAT a; cdc31-1 rrm3:: KanMX* | This study |
| WDHY3661 | W303-*RAD5 MAT α; cdc31-1 rrm3::KanMX* | This study |
| WDHY5102 | W303*-RAD5 MAT a; mus81::KanMX* | This study |
| WDHY5293 | WDHY3006; *est2::ura3::LEU2/EST2 mus81-D414,415A-9Myc::TRP1/MUS81 rad51::LEU2/RAD51* | This study |
| WDHY5295 | WDHY3006; *est2::ura3::LEU2/EST2 mus81-D414,415A-9Myc::TRP1/MUS81 rad59::LEU2/RAD59* | This study |
| WDHY5296 | WDHY3006; *est2::ura3::LEU2/EST2 MUS81-9Myc::TRP1/MUS81 rad51::LEU2/RAD51* | This study |
| WDHY5297 | WDHY3006; *est2::ura3::LEU2/EST2 MUS81-9Myc::TRP1/MUS81 rad59::LEU2/RAD59* | This study |
| WDHY5327 | *MAT a/α; ade2-1/ade2-1 can1-100/CAN1 his3-11/his3-11 leu2-3,112/leu2-3,112 ura3-1/ura3-1 trp1-1/trp1-1 est2::ura3::LEU2/EST2 mus81::KANMX/MUS81 rad5-G535R/ rad5-G535R* | This study |

All strains are W303 background with corrected RAD5 and were constructed for this study unless otherwise noted.

* Strains generously provided by Rodney Rothstein (Columbia University)

^ǂ^ Strain generously provided by Stephen Elledge (Harvard University)

^ Strain generously provided by Adam Bailis (Beckman Institute)

^ǂǂ^ Strain generously provided by Steven Brill (Rutgers University)
