# Supplementary material for: Saccharomyces cerevisiae Mus81-Mms4 prevents accelerated senescence in telomerase-deficient cells
Source: PLoS Genet. 2020 May 29;16(5):e1008816. doi: 10.1371/journal.pgen.1008816 (PMC7286520; doi:10.1371/journal.pgen.1008816)
Supplement: Attachment — Submitted filename: Response to Reviews.pdf [file pgen.1008816.s014.pdf]

Response to reviews

We want to thank both reviewers for their constructive criticism that allowed us to substantially strengthen the manuscript by adding new experimentation and clarifications. These revisions address the concerns of the reviewers. The detailed responses to the reviews and explanations what changed in this revision are listed in blue after the reviewer's comments. The major additions are:

1) The key technical concerns of Reviewer #1 were fully addressed. First, our representation of the variability was more transparent than in other publications. We now show our data in a way that is consistent with those previous publications, which substantially tightened the error bars. Second, the reviewer was worried that 2 days of incubation distorted our data, but we show experimentally that there is no difference between 2 and 5 days of incubation.
2) ChIP data (comment 2, Reviewer #1; comment 8, Reviewer #3): We have significantly expanded the ChIP analysis and added the requested controls, as well as ChIP experiments in survivors including type I and type II survivors. Figure 5 (previously 7) was significantly expanded, new Figure S5 was added.
3) Genetic analysis (comment 3, Reviewer #1; comments 6, 9, Reviewer #3): We greatly expanded the genetic analysis adding Figures 4B, 6A-D, S6, S7.

Reviewer #1: This manuscript (re)visits the role of the Mus81-Mms4 endonuclease in telomerase-independent growth in budding yeast. In contrast to a prior report, this study reports that the absence of Mus81-Mms4 results in an accelerated senescence in response to a telomerase deficiency, although Mus81-Mms4 is not subsequently required for the appearance telomerase-independent survivors. A ChIP experiment shows that Mus81 localizes to telomeres (which unfortunately is only examined in a telomerase-defective background). Epistasis analysis was used to show that the modest sensitivity to HU and MMS displayed by a mus81 mutant was further enhanced by as cdc13-1 ts mutant as well as a defect in the Rrm3 helicase.

Collectively, the authors would like to argue from this somewhat disparate set of observations that they have uncovered a new role for Mus81-Mms4 in "recovery of stalled replication forks within telomeric DNA", and furthermore, this endonuclease acts "facilitates telomere replication during times of telomere stress" (both quotes taken from the abstract). It's an appealing model, but the data fall short of providing strong support.

General comments:

1. The most substantial concern is that the results reported here dispute a prior study by the Johnson lab, which also tested mus81 in a liquid senescence assay and found no statistically significant difference between MUS81 and mus81 strains. A subsequent genome-wide study by Lydall's group recovered numerous genes required for replication fork progression, but not MUS81 or MMS4. One could argue that the genome-wide study simply missed these two genes, but it is harder to argue against the direct test by Johnson and colleagues. The authors state that their result is correct, and Johnson's group was wrong, because of a difference in the protocol that they employed. I would argue the opposite; as described in detail below (under technical

comments), I feel that this current study made a substantial error in how viability was measured during liquid growth of telomerase-null strains.

The technical concerns and differences to the Johnson laboratory are addressed below, where the reviewer details the technical comments.

The reviewer also mentions the work by the Lydall lab, and highlights that their screen for est1-del genetic interactions did not identify *MUS81* or *MMS4* (Chang et al 2011 Genes Genomes Genetics). However, Lydall's group themselves states on pg 205 of their manuscript that, "Some previous data agree with our results whereas other data do not; it is not clear whether the differences are due to strain background or some other factor, such as experimental design." Lydall's group performed their high-throughput screen with solid growth medium, whereas our study was performed in liquid. Differences between solid and liquid growth media are already known to affect the selection of yeast survivors (Teng and Zakian 1999 MCB) and even within their study Lydall and colleagues identified instances, such as *rad52 est1*, where they, "interestingly showed different growth patterns in solid *vs.* liquid plates." We should note that Lydall's group did perform a medium-throughput screen in liquid media, but did not include *mus81* or *mms4*.

2. The Mus81 ChIP data: a critical missing control was the Mus81-telomere ChIP analysis in telomerase-proficient cells. If Mus81 shows the same extent of association with telomeres in the presence of telomerase, then it is hard to argue that Mus81 is acting in response to replication stress at telomeres. And given that this study is driven by prior observations showing that Mus81 is required for proliferation of human ALT cells, it was surprising that association of Mus81 in telomerase-independent survivors was not monitored. As an aside, using a mus81-del strain as the "control" is less than ideal (particularly given the author's claim that this strain has accelerated senescence); the control should be a strain that lacks the myc tag on Mus81 but is otherwise isogenic to the experimental strain. Overall, the ChIP experiment, as currently designed, did not provide strong support for the premise of this study.

We appreciate the reviewer's suggestions and have completed all proposed ChIP experiments. The undetectable levels of Mus81 at the telomere with wild type *EST2* (updated Fig. 5) supports our model that Mus81 is recruited to telomeres during times of telomere stress. Analysis of Mus81 enrichment in survivors shows a reduced association (Sup. Fig. S5), suggesting the role for Mus81 may be in shortening telomeres as supported by other groups. Finally, we have included additional negative controls of untagged Mus81 and Mus81-dd mutant in the updated Figure 5 which, as expected, show no enrichment over the no-antibody control. We have also added the analysis of telomerase-independent survivors in Figure S5.

3. Re the epistasis analysis: the mus81 cdc13-1 result is intriguing, but the impact of the mus81 rrm3 epistasis less so. The authors state that Rrm3 is a "known telomere replication factor" (from the abstract), but this is a claim that most in the telomere field would question. In the absence of Rrm3, replication forks are indeed more susceptible to stalling and pause more frequently in sub-telomeric regions. However, this has no functional consequence for telomere biology: for example, in a telomerase-null strain (which the authors would like to argue presents a situation which "creates replication stress), loss of Rrm3 has no phenotypic consequence on the growth of a telomerase-defective strain, an observation which is published by the Johnson lab and also repeated in many labs since the original publication (including our own lab). In

contrast, an rrm3 defect is either synthetically lethal (sgs1, mrc1, srs2, mrx) or synthetically sick (chk1, mec1, rad53, rad9) with genes that also have a genetic interaction with Mus81. Thus, the mus81 rrm3 phenotype does not support the conclusion that " Mus81 and Rrm3 function in independent or only partially overlapping pathways to support telomere replication forks", as the authors propose.

If loss of Rrm3 (a known factor that contributes to telomere replication) has no functional consequence in growth and viability under conditions of telomere and replication stress, then it would be indeed hard to argue that the phenotypes we are observing in the absence of Mus81 would be due to defects in telomere replication. To explore the contribution of Rrm3 and Mus81 in the context of telomere stress, we performed two additional experiments. First, we evaluated the growth and viability of single *mus81* and *rrm3* mutants and combined double mutant with functional and compromised Cdc13. These results show clear synergistic effects in the *rrm3 mus81* double mutant relative to either single mutant deletion in the *cdc13-1* background (new Fig. 6d), showing the importance of both proteins in situations of telomere dysfunction. This synergistic growth defect is even more pronounced in the presence of replication stress (new Fig. 6d). Furthermore, the *rrm3 mus81* double mutant alone shows only a modest reduction of growth in the presence of replication stress, further supporting that both Mus81 and Rrm3 are contributing to replication support specifically in the context of the telomere. Second, we have performed senescence assays in both single mutants and *mus81 rrm3* double mutants and show that although loss of *RRM3* alone does not cause increased senescence in the absence of telomerase, in the absence of *MUS81*, the importance of Rrm3 becomes apparent as the triple mutant is now severely affected (Fig. 6a-c).

The technical concerns about the senescence assay:

First, a general point: the senescence phenotype has a significant sample-to-sample variation, which makes statistically robust comparisons between genotypes very challenging, regardless of the assay used. This point well-illustrated in the primary data provided by the authors in Supplem Fig. S1. For example, in part a of Fig. S1, at the ~43 generation timepoint, the viability of the five est2 mus81 clones spans a 100-fold range (from 1.0E+07 to 1.0E+05): at the ~55 timepoint, there is a greater than 100-fold range in viability for these five clones. This argues that there must be a huge standard deviation for at least some of these time points. Notably, all of the senescence assays shown in Figures 1, 2 and 4 lack error bars, which is an omission that is even more conspicuous given that error bars were shown in Figure 5. For these reasons alone, I am not convinced that there is a statistically significant difference among the various genotypes shown in Figures 1, 2 and 4.

Sample-to-sample variation is indeed a challenge with this assay as the strains are senescing and recovering at different rates. In addition, slight changes in aeration or media can cause changes over a 14-day assay. This is why we had highlighted this point with previous Supplemental Figure S1. Most groups simply plot cell concentration without any error bars (LeBel et al. 2009 Genetics, Grandin and Charbonneau 2003 MCB) or combine all spore clones and plot the average and standard error of the mean (Azam et al 2006 NAR, Meyer and Bailis 2008 PLoS ONE). We had discussed that both methods do not fully demonstrate the variability of this assay. To minimize the effect of clonal variation and run-to-run variation, we decided to always analyze 8 spore clones of each strain, included wild type and *est2* controls with every serial dilution experiment, and only compared strains from a single experiment. However, in response

to the concerns of the reviewer, we have combined the data from all spore clones and plotted their average with a single standard error as suggested. This does present the data in a manner that is more comparable to the previous studies, but it is difficult to appreciate the assay variability.

The reviewer's concern that the viability plots in previous Supplemental Figure S1 are all independent clones is due to a mistake in the key. Rather than individual clones, the plots were all median values of ~8 spore clones analyzed at different times (representing over 30 individual clones for *mus81 est2*). We have since combined all individual clones for analysis and plotted their average and standard of error as shown in the new figures. The concern about the 100-fold range at ~43 and ~55 generations is due to run-to-run variation clearly observed also by other groups including Johnson and colleagues (Johnson et al 2006). Comparing their *tlc1* control between experiments in Figure 3 of their publication, the number of generations to obtain lowest cells/ml was observed between 60 and 85 generations. Although trends are the same, the timing of senescence and recovery were different, likely due to media, temperature, or aeration differences, which is why we previously compared only strains tested together at the same time.

To address the concern about the error bars, we have replotted all of the data as averages with a single standard error similar to other publications (Chen et al 2001 MCB, Azam et al 2006 NAR, Meyer and Bailis 2008 PLoS ONE). It should be mentioned that the error bars in Figure 5 was the standard deviation of the viability data as described in the figure legend and is not comparable data to the serial dilution viable cells/ml quantification. For consistency we also replotted the viability with standard error.

Now that the data is presented in a way that shows standard error, we still show statistically significant differences between different genotypes in regards to their growth and viability, as shown by student T-test analysis in the text and Figures 1d, 2b, 6b and 6c, Supplemental Figure S2b.

However, there is a larger problem, which has to do with how the authors measure the decline in viability in telomerase-null cultures: they define viable cells, in Figures 1 and 5, by the appearance of colonies after exactly two days, and this is a huge technical error. The authors are only surveying one particular (healthy) sub-population; i.e. those cells that have a cell division rate fast enough to give visible colony after 48 hours. However, cells with only a slightly cell division will not be counted in this protocol; for example, if cells undergo just ~3 fewer cell divisions during the growth of a colony, this will result in a colony size that is 10-fold smaller after 48 hours. These slower growing cells are viable and contribute to the growth of the culture, but they are invisible in the assay the authors use. This is also why the prior publication from the Johnson lab (reference 59 in this current study) did their senescence assays by total cell counts; also not ideal, because inviable cells are included, but at least in this latter assay, every viable cell is measured.

To address the reviewer's concern, we have plotted our graphs with cell density to be more consistent with the Johnson Lab. This concern about colony formation is something that we explored. In our methods it states that we counted colony-forming units after, "… at least two days". Plates were grown for exactly two days for the WT strains, which showed no viability defects, and up to 5 days for est2-del strains. However, in early serial dilution assays we looked

at the colony-forming units at 2 days and at 5 days and saw that the numbers of identified colony forming units were identical between these two time points, and often the senescing colonies did not grow much after day 2 (see quantification below). We mention this now specifically in the description of the procedures in Material and Methods as data not shown. We show the data below, but did not include them in the manuscript, unless the reviewers or the editor think this is helpful.

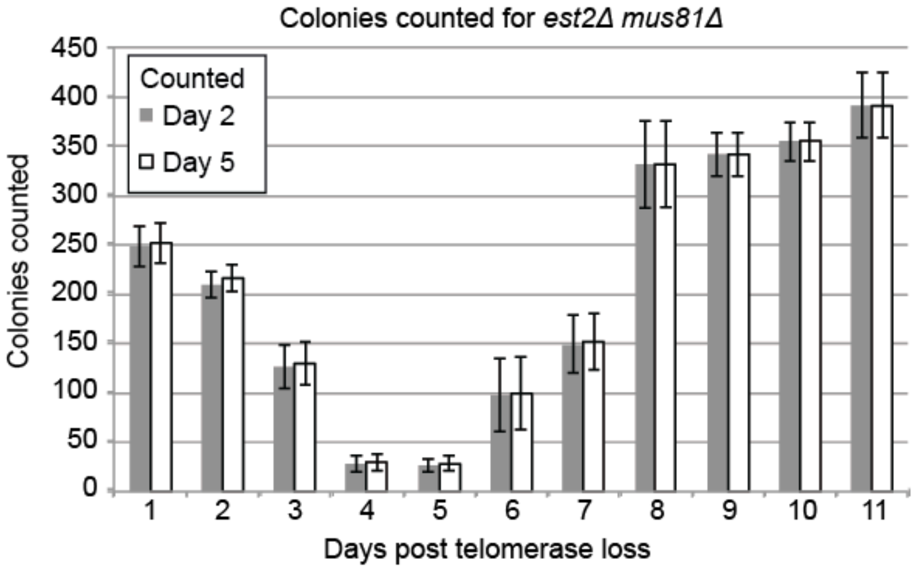

In the past, individuals have used only cell density to monitor cell growth (Azam et al 2006 NAR). However, considering the significant viability difference between *est2* and *mus81 est2*, we believe that taking viability into account is an important factor to monitor in this experiment, and in fact should be adopted by others in this field.

As we discussed in the text, results from the Johnson lab did not observe a senescence phenotype with *mus81 tlc1* (Azam et al 2006 NAR). However, we believe that incorporating viability data is important for these studies, as it provides additional information about the senescence phenotype. Given that the difference in viability between *est2* and *mus81 est2* are statistically significant for the first 50 generations (Fig. 5b), it is not surprising that incorporating this information results in an overall difference in viable cell density. This difference in viability is completely unaccounted for in the study by the Johnson laboratory, and could account for the differences between these studies. Furthermore, our robust analysis (with over 30 independent spore replicates for *mus81*) identified reproducible phenotypes for both *mus81* and its partner Mms4, and also found the growth phenotype to be dependent on catalytic activity of Mus81.

Minor:

The manuscript is over-long in every section (particularly the discussion!), with multiple repetitions of many observations; it could benefit from substantial editing throughout.

We have made efforts to shorten all sections and limit the discussion section.

page 17: yeast does not undergo "crisis" during replicative senescence (crisis in mammalian cells is a p53-dependent process that allows cells undergoing replicative senescence to bypass a critically short telomere length barrier and continuing dividing).

The term "crisis" has been used more carefully to describe the growth and viability crisis that occurs during senescence as used recently in Nature Communications (Millet et al 2015).

Missing in M&M is one key detail re the senescence assay: how were cells diluted after each 24 hour time point? (since the authors were not determining total cell counts, it is unclear how this step was executed).

We determine the total cell counts to reseed the next inoculation and calculate the number of cells for plating and thus the viable cell densities. We have since added the mechanism of determining cell density, "by cell counting" and a line was added to the materials and methods on pg 27, "After 24 hours total cell bodies were counted and this value used to inoculate the next overnight culture and plating for viability."

The analysis of the potential role for Mus81 in the survivor pathway was not particularly rigorous. Figure 3 shows the telomere profile from 5 independent isolates, from a timepoint past 80 generations. There was no information describing how these 5 isolates for each genotype were generated, but extrapolating from the M&M and experimental approaches used through the rest of the paper, I'm guessing that these isolates were recovered after liquid propagation, rather than by serial streak-outs. If so, this does not provide any information on the presumed role of Mus81 in the process, because Type II survivors have a selective advantage in liquid media. In addition, 5 isolates are insufficient to make any conclusions. See the Teng et al. Zakian Mol Cell 2000 paper for experimental techniques (and appropriate numbers of isolates) necessary to answer the question of whether Mus81 has an impact on the frequency of Type I vs. Type II survivors.

The question that we set out to answer was if Mus81 was REQUIRED for either Type I or Type II survivor formation, not if it affected the frequency of survivor formation. Based on analysis of human MUS81, we predicted Mus81 was required for Type II survivors, which are mechanistically similar to human ALT. We do not make any conclusions about the effect of Mus81 on survivor frequency, as it was not something that we set out to determine, and we do consider this to be of importance in the context of this manuscript.

With regards to the rigor of how we analyze the requirement of Mus81 in survivor formation, we provide two conclusive sets of data that includes genetic (Fig. 3) and physical (Sup. Fig. S3d) methods to explore the role for Mus81 in Type I and II survivor pathways. In Figure 3, we show genetically that Mus81 is not required for either Type I or Type II survivor formation, unlike other recombination components (*e.g.* Type I: Rad51; Type II: Rad59 and Sgs1) (Chen et al 2001 MCB). To clarify this genetic point, we have included a cartoon diagram about the genetic requirements of each pathway to further explain our reasoning. As a complementary experiment, in Supplemental Figure S3d we show that Mus81 is not required for Type II formation, as they readily form in the *mus81 est2*. It also shows that the survivor telomeres in a *mus81 est2* look visibly similar in structure to *est2* alone.

As to how the isolates in Figure 3 were generated, we have included information about their construction in the figure legend and material and methods (pg. 28). “Five independent clones were selected after tetrad dissection and independently processed for serial dilution analysis. At approximately 25 and 80 generations, cells were pelleted and DNA extracted for Southern blot. ”

We are also aware that Type II are advantaged in liquid culture and have already mentioned this in the manuscript as an expected outcome for the *est2* strain (pg. 12). “In the absence of telomerase, it has been observed that Type I survivors occur first, and then are soon outcompeted by Type II survivors resulting in a predominately Type II survivor population at later generations times (>80)”

Reviewer #3: In this MS, Schwartz et al. evaluate the contribution of the budding yeast Mus81-Mms4 in telomerase negative cells (est2Δ). They show that in the absence of Mus81-Mms4 activity, est2Δ cells exhibit accelerated senescence and produce type II survivors. They infer from the analysis of est2Δ mus81Δ rad51Δ and est2Δ mus81Δ rad59Δ that Mus81 does not affect type I or Type II telomere recombination. They conclude from the telomere binding of a catalytic deficient Mus81 in est2Δ cells and the increased sensitivity to HU and MMS of the double mus81Δ cdc13-1 compared to each single mutant thatMus81 is required for telomere replication particularly in the presence of telomere replication stress. Finally, by combining mus81Δ with rrm3Δ and analyzing the sensitivity of the double mutant in MMS and HU, they propose that Mus81 and Rrm3 independently stabilize replication forks in response to DNA replication stress.

Understanding the detailed roles of Mus81-Mms4 in telomere replication and maintenance is of great significance and interest, given the importance of Mus81-Mms4 in genome integrity. However, the submission of the work is really too premature. Many important experiments are missing to conclude about the role of Mus81-Mms4 in telomere replication either in est2Δ cells or under conditions of telomere replication stress. Moreover, some experiments aren't convincing or do not support the conclusions. which together make for a rather confusing story.In general, it is not easy to follow how the authors link the effects associated with mus81Δ in telomerase negative cells with those observed under conditons of replicative stress.

I have listed a number of points that should be useful for the authors to improve their MS.

1) In Figure 1, it is essential:
a) To plot the variability of the senescence kinetics
b) To do a graph showing cell density at crisis peak for each mutant
c) and most importantly- for the est2Δ, est2Δ mus81Δ, est2Δmms4Δ and est2Δ mus81-dd – to perform for each of the different time points of the senescence kinetics a Southern blot (XhoI-digestion) probed with a TG1-3 probe.

1a) We included this in the new analysis.
1b) We constructed a figure at the request of reviewer #3 (below) and have included it as an extra panel in Figure 1.

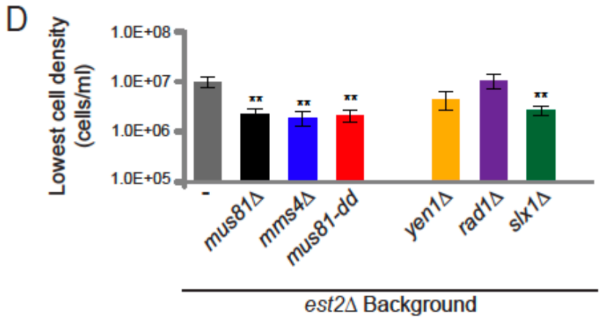

1c) As mentioned in the last response to reviewer #1, the question that we set out to answer in Figures 3 and 4 was if Mus81 was REQUIRED for either Type I or Type II survivor formation, not if it affected the frequency of survivors. Based on analysis of human MUS81, we predicted Mus81

was required for Type II survivors, which are mechanistically similar to human ALT. The results answered this question unambiguously. We do not make any conclusions about the effect of Mus81 on survivor frequency, as it was not something that we set out to determine. See question #4 for additional details about the assays used. Moreover, it is not clear, if such an experiment is feasible, as in an *est2* mutant telomere shortening already occurs by day 2.

2) Figure 1A, 1B and Fig. 2B and 2C should be merged in Fig. 1A and Fig. 1B.

We thank the reviewer for this suggestion and plotted them below.

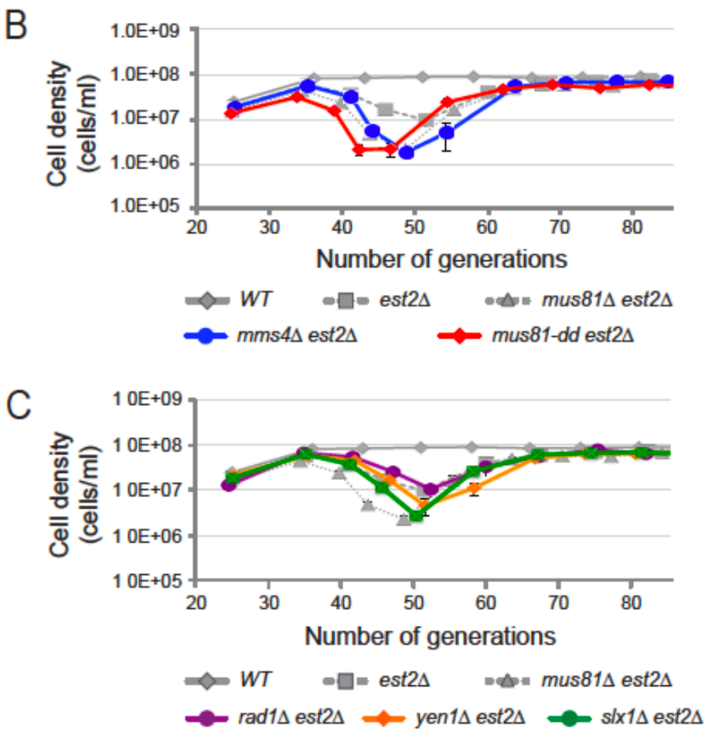

3) Figure 3 shows that est2Δ mus81Δ cells do type II survivors but it does not tell whether the kinetics of Type II formation is affected (see point 1). In Figure 3 the bands are very faint. Why not using a Y' probe rather than oligonucleotides?

As mentioned in response to comment 1c, the question that we set out to answer was if Mus81 was REQUIRED for either Type I or Type II survivor formation, not if it affected the frequency of survivors. In Supplemental Figure S3e we establish that Mus81 is not required for Type II survivor formation, as they form readily in *mus81 est2* survivors. We have increased the contrast for the bands in Supplemental Figure S3e so that the higher molecular weight bands clearly identify the pattern for Type II survivors.

As diagramed in Figure 5a, as well as mentioned in the figure legend and in the methods, we are using a Y' probe in Figure 5b.

Figure 5b diagram of probe location on Y' element (see black bars).

D

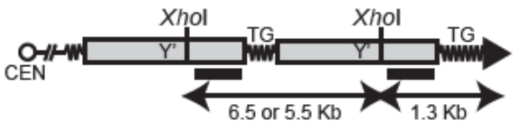

Supplemental Figure S3d legend, "Southern blot analysis using the Y' probe can monitor both telomere repeat length and the status of the Y'-elements [85]…. Genomic DNA was probed using an oligonucleotide complementary to the Y'-element region adjacent to the telomere indicated in (B)."

Materials and Methods (pg 28), "The DNA was transferred onto nitrocellulose using capillary action and blotted with probes to the Y'-element (Fig. 3, B). The probes for Southern analysis were prepared using PCR with primers T3 (5'-AGC GCG CAA TTA ACC CTC ACT AAA G-3') and T7 (5'-CGT AAT ACG ACT CAC TAT AGG G-3') with pRS313/Y' *Rsa*I template, kindly provided by Dr. Gottschling [85]."

We utilized the Y' probe due to the ability to focus on both the Y' elements (which are preferentially amplified in Type I) and also the TG_{1-3} repeats (shorter band signal). The blots appeared cleaner than our TG_{1-3} probes.

4)In Figure 4
a) To show that Mus81-Mms4 is not essential for either Type I or Type II survivors in telomerase-deficient cells, you must show a Teloblot (Southern) showing that est2Δ mus81Δ rad59Δ cells form type I survivors with the same kinetics as est2Δ rad59Δ
b) Alternatively, the authors can use another mutant affecting Type II pathway and combine it with est2Δ mus81Δ
c) It is informative for each mutant to make a graph showing the duration of the crisis. The duration of the crisis looks to increase in est2Δ mus81Δ rad59Δ suggesting that mus81Δ does affect type I.

We do not quite understand the point, as the reviewer lists under b) exactly the approach that we took. In Figure 3c we use a mutant of Rad59 (that is required for Type II formation) to create a situation that only produces Type I survivors (Chen et al 2001 MCB). Therefore, if Mus81 was required for Type I survivor formation, then there should be no growth in the combined triple mutant, *mus81 est2 rad59*. However, survivors readily form in the triple mutant suggesting that Mus81 is not required for Type I formation. A similar argument can be made for the *rad51* genetics and Type II survivor formation. This genetic method of identifying components of Type I and Type II survivor pathways is established and has been used to previously identify numerous components of these pathways (Chen et al 2001 MCB, Lundblad and Blackburn 1993). We have included a cartoon diagram in the figure to better illustrate our experimental design and predictions (Fig. 3a).

4c) By plotting the entire serial dilution up to 80 generations, we show both the decline and recovery of viable cell density. The challenge in defining "duration of the crisis" is that there is no clear distinction of the beginning and end of this dynamic period. The cells are already undergoing a reduction in cell viability and growth before the first 24-hour time point, and the

24-hour time interval (~7 generations) does not accurately define the exact point of recovery. If the reviewer has additional suggestions on the best way to perform this analysis in an accurate and objective manner, we would be happy to create the additional figure. There is indeed a reproducible difference in duration under some genetic conditions and we have included a line in the text describing the possibility that Mus81 may contribute to Type I formation on pg 11, “Slight deviations in the timing and duration of cellular senescence and recovery were observed in the absence of MUS81 and may suggest a partial role in these pathways.” and on pg. 20 “Changes in the timing of cell density crisis in est2Δ mus81Δ rad51Δ and est2Δ mus81Δ rad59Δ triple mutants (Fig. 3, B and C), suggests a partial involvement of MUS81 telomerase-deficient survivor formation, but not an essential role as predicted.”

5) Figure 5 is redundant with Figure 1. Again it is informative to plot cell density at crisis.

Figure 2 is specifically looking at viability, not viable cell density (a value derived from both viability and growth data). This figure focuses our attention on the effects of Mus81 specifically on the viability of telomerase-deficient cells. To clarify this point, we have added additional labels in the figure legend and text, pg. 9, “Interestingly, the est2Δ mus81Δ double mutant exhibited a 56% reduction in cell viability compared to the *est2* mutant alone (t(64)=2.94, p-value=0.0045, Fig. 2, B).”, “This significant reduction in viability was unique to Mus81, as it was not observed for any of the other endonucleases (Fig. 2, B)”

As mentioned in the previous comment, we have included the cell density and viability at crisis for these graphs as well and have included it as the graph below in Figures 1d and 2b.

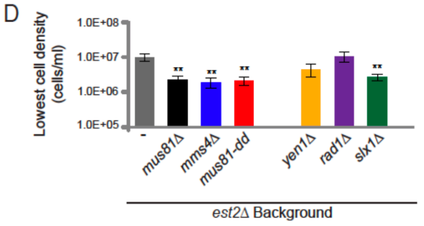

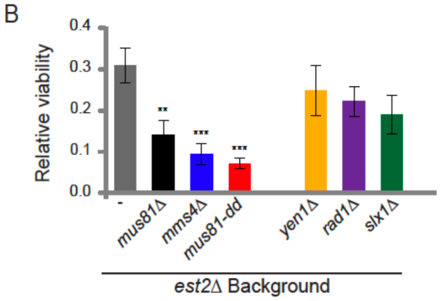

6) cdc13-1 cells accumulate large extends of telomeric ssDNA, a very different situation than est2Δ cells. How the effects of Mus81 in telomerase negative cells are linked to those under replicative stress.

This is an excellent point. We proposed that shortening telomeres and telomere uncapping could present opportunities for replication stalling, in addition to the general challenges with telomere replication (reviewed in Gilson and Geli 2007). We have since completed viability and growth analysis of telomerase negative cells during early senescence and "crisis", showing that est2-del alone is sensitive to the replication stalling agent methyl methanesulfonate, and additional loss of *MUS81* leads to a synergistic loss in viability and growth in the presence of the replication fork stalling agents HU and MMS (Fig. 4B).

7) To verify that Mus81 affects telomere replication in WT and overall in est2Δ cells, 2D-gel analysis must be performed. This type of experiment is essential for most of the conclusions of this study

We agree that seeing differences in replication fork stalling by 2D-gels would be an excellent addition to this manuscript and direct evidence for our model. We have performed 2D-gel analysis in *mus81* and *rrm3* single and double mutant strain backgrounds at a subtelomeric region previously shown to have *Rrm3*-dependent replication fork arrest (Ivessa et al 2002 Genes Dev). Unfortunately, the *Rrm3*-dependent fragile sites showed no difference in stalling, with or without Mus81 (Sup. Fig. S6d-f). Although we do not see evidence of stalling at these particular sites, it does not eliminate the possibility that Mus81 is affecting general replication fork stalling at subtelomeric regions, nor that Mus81 plays a role in replication fork stability in telomere repeats.

At this point, our model that Mus81 is stabilizing replication forks at dysfunctional telomeres is supported by molecular and genetic evidence. First, Mus81 is recruited to telomeres specifically during telomere crises in the absence of telomerase. Second, *est2* cells exhibit reduced viability in the presence of replication stalling agents. Third, *cdc13-1* cells are only sensitive to replication stress at semi-permissive temperatures, suggesting that telomere dysfunction does result in replication defects (Fig. 4). Loss of Mus81, Rrm3, or both, under conditions of mild replicative stress results in severe growth and viability defects ONLY when combined with dysfunctional telomeres (new Fig. 6). This suggests that defects at the telomere renders cells more susceptible to replication stress, and that Mus81 and Rrm3 are required for growth and viability under these conditions. We show this for both conditions of telomerase loss and telomere uncapping.

8) Binding of the Mus81-dd-Myc to telomeres is interesting. Does the Mus81-dd-Myc bind to telomeres in EST2+ cells? Non-tagged Mus81 is the best control to monitor the binding of Mus81-Myc at telomeres in est2Δ cells. The authors may look for conditions that improve the telomere binding of the WT Mus81-Myc protein whose binding appears barely significant.

We have performed the appropriate ChIP experiments to evaluate Mus81 and Mus81-dd levels at the telomere under normal growth conditions. The undetectable levels of Mus81 at the telomere with wild type EST2 (updated Fig. 5) supports our model that Mus81 is recruited to telomeres during times of telomere stress. We have also included additional negative controls of untagged Mus81 and mus81-dd mutant in an updated Figure 5 which, as expected, show no enrichment over the no-antibody control. Our evaluation of Mus81 enrichment to telomeres in telomerase-negative survivors, surprisingly showed no enrichment over wild type EST2 conditions (new Sup. Fig.S5).

9) Figure 8 should further documented beyond the synthetic sickness between mus81Δ and rrm3Δ

We appreciate the suggestion and have performed three additional experiments to investigate the synthetic sickness between *mus81* and *rrm3*. First, we performed a quantitative viability assay to better quantify the synthetic sickness between *mus81Δ* and *rrm3Δ* under normal conditions and conditions that induce replication stalling (Sup. Fig. S6). Second, we completed additional viability assays to evaluate their functional interaction under situations of telomere uncapping (*cdc13-1*) and telomerase loss. These results are included as additional panels in the new Figure 6.

Minor comments:
- Page 5 correct metagbolism by metabolism

Done.

- Figure 3A. Type I recombination is initiated by recombination between the terminal telomeric repeats of the shortened telomere and the internal telomeric tract located between subtelomeric elements (Lundblad and Blackburn, 1993, Churikov et al. 2014).

We have updated the figure to clarify this point.
