# Supplementary material for: Saccharomyces cerevisiae Mus81-Mms4 prevents accelerated senescence in telomerase-deficient cells
Source: PLoS Genet. 2020 May 29;16(5):e1008816. doi: 10.1371/journal.pgen.1008816 (PMC7286520; doi:10.1371/journal.pgen.1008816)
Supplement: Attachment — Submitted filename: Response to reviews_2020.docx [file pgen.1008816.s015.docx]

**Response to reviews**

We would like to thank the three reviewers for their helpful evaluation of our manuscript. Their constructive critiques allowed us to strengthen our manuscript by adding new experimentation and clarifications. These revisions were completed to directly address the concerns of the reviewers. Detailed responses to the reviewers and explanations of what has changed in this revision listed in blue after the reviewer’s comments. The major additions are as follows:

1) We changed the title as suggested by reviewers 1 and 3.
2) An entirely new serial dilution assay with *tlc1∆ and* *mus81∆*. was added/
3) Additional genetic analysis using *mus81∆* full deletion to make Supplemental Figure S6 more consistent regarding the genetics was added.

Additional clarifications were made in regards to how terminology was used and additional alternative interpretations included where appropriate.


**Authors’ Responses to Reviewer’s Questions**

Reviewer #1: Schwartz et al present evidence that Mus81-Mms4 plays a role in promoting cell viability during telomere-induced senescence. They also show that Mus81 is not required for Type I and II survivors to form. Based on their findings, Schwartz et al propose that loss of telomerase causes replication stress at telomeres, which Mus81-Mms4 is needed to deal with. This is certainly a plausible idea that could account for their data. However, I think that their body of evidence falls a bit short of what would be required to definitively support the bold claim that the title of their paper makes – “Mus81-Mms4 endonuclease is required for telomere maintenance under replication stress in Saccharomyces cerevisiae”. I think they would have a much stronger case if they showed that Mus81 was exclusively enriched at telomeres in telomerase defective cells (e.g. by a calibrated ChIP-seq experiment). As it stands, one could propose that profound changes in gene expression, which are known to occur during cellular senescence, could lead to genome-wide problems that may require Mus81’s attention.

We agree with Reviewer #1 and changed the title to: ”Mus81-Mms4 prevents accelerated senescence in telomerase-deficient cells.” as also proposed by reviewer #3.

Other comments:
1) Figure 2: The data appear to show that Mus81 is required for the viability of Type I/II survivors (i.e. from ~65 generations onwards the viability of mus81∆ est2∆ cells is less than est2∆ cells). The authors should make a comment about this.

We appreciate the reviewer’s suggestion and we now mention this observation in the results section.

p. 10, Line 1 – “Upon recovery, the viability of *est2Δ* is equivalent to wild type (t(11)=1.51, P=0.1592, Fig. 2A). However, we observed a 14% reduction in viability of survivors with additional loss of *mus81* compared to *est2Δ*  alone (t(8)=5.75, P=0.0004, Fig. 2A).”

The authors should also include data for the relative viability of a mus81∆ single mutant to confirm that it doesn’t differ from wild-type by the same order of magnitude as a mus81∆ est2∆ double mutant differs from an est2∆ single mutant.

We had previously performed this control and saw no difference in relative cell viability between wild type and *mus81∆* (S7 Fig.). We now include new data in the new S6 Fig., which shows no difference in the cell concentrations compared to the wild type control. Appropriate text updates were also included.

2) Figure 2: The data in panels A and B don’t appear to be consistent – in A the lowest relative viability for the est2∆ mutant is ~0.26 – 0.27, whereas in B it is shown to be 0.3.

The reviewer points out an important distinction between these two data sets, which we should have clarified. Panel A is reporting viability across all generations, whereas Panel B is reporting cell viability specifically at crisis (as defined by the lowest cell concentration in Fig. 1). It happens that the cell viability at crisis does not always have the lowest viability. The language of the caption has been expanded and additions to the y-axis label have been made to be more explicit about this important distinction. We clarified this distinction also in the text.

Figure 2 caption

p. 42, line 17 - “(*B*) Relative cell viability was calculated at the generation with the lowest average cell density (senescence crisis).”

3) I think that the authors should do more to establish why their data differs so markedly from previous studies. At the very least, they should establish whether this is due to a difference between est2∆ and tlc1∆.

In response to the reviewer’s concerns we have included new data in S7 Fig. that report the results of the requested experiment with *tlc1∆* in this serial dilution assay. The results are fully consistent with the *est2∆* genetics. We went further to explore the effect of the combination of *tlc1∆* and *rad5-535* (another discrepancy between the two studies). The enhanced crisis in the *mus81∆* with or without functional *RAD5* is consistent with the *est2∆* data.

4) There are a few spelling/grammar/typographical errors, e.g. page 19 “platting” and supplemental page 11 “Average fold-enrichment of with three replicates..”

We thank the reviewer for pointing out these text errors and corrected them.

Reviewer #2: Previous studies in human cells suggested a role for MUS81 in extension of telomeres by a recombination-based mechanism, referred to as ALT. In this study, the authors use budding yeast to investigate whether the function of Mus81 in the generation of survivors from telomerase-negative cells is conserved, and to assess whether Mus81 has a more general role in telomere biology.

The authors find that survivors arise from est2 mus81 cells with similar kinetics to est2 cells. Moreover, by use of rad51 and rad59 derivatives of est2 mus81 cells, the authors show that Mus81 is dispensable for the generation of type I or type II survivors. Consistent with these observations, Southern blot analysis of survivors from est2 mus81 showed the typical type II pattern with long, heterogeneous telomeres.

In contrast to previous studies (Azam et al, 2006; Fallet et al, 2014), the authors report accelerated senescence and reduced viability of est2 mus81 cells as compared to the est2 single mutant, which they attribute to a role for Mus81 in resolving replication stress at telomeres. In support of this idea, the authors show that mus81 enhances the MMS and HU sensitivity of est2 and cdc13-1 mutants, and Mus81 in enriched at telomeric DNA in pre-senescent cells. The authors also report additive defects of mus81 and rrm3 in the est2 or cdc13-1 background.

These observations raise the question of whether the requirement for MUS81 in ALT is related to recombination or to the increased replication stress in ALT cells. Thus, the work will be of interest to investigators studying ALT, as well as the structure-selective endonuclease field.

Comments:

I am not completely convinced that cdc13-1 mus81 null cells have reduced viability compared with cdc13-1. Were cell division times calculated? Also, because of the decreased viability of the cdc13-1 mus81 rrm3 triple mutant at 27 C in the absence of MMS, I don’t think the authors can conclude that the triple mutant is more sensitive than the doubles to MMS. They would need to do quantitative survival assays to make this claim.

Serial dilution spot assays invariably report on growth and viability which cannot be separated without independently testing each component. We have, therefore, updated our text to strictly reference the observed spotted cell density and colony size. (p.14 lines 1-2, 5, 18; p. 17 lines 4-5).

We also highlighted the specific plate and condition from where our claim is based.

p. 14 line 16-18 - “Importantly, both double mutant combinations, *cdc13-1 mus81Δ* and *cdc13-1 mus81-dd*, resulted in a severely reduced spotted cell density and colony sizes at the semi-permissive temperature in the MMS-treated condition and a moderate reduction in the HU-treated condition (Fig. 4C).”

Do mus81 null rrm3 spores have smaller colony sizes? Some figures show the mus81-dd allele and others the null. In the case of synthetic interaction with rrm3 the authors should be consistent with which allele they use in different assays.

As requested, we have repeated the crossings to include the full *mus81* deletion mutant with *rrm3*, which showed reduced size of the double mutant compared to either single mutant although not to the extent as the catalytic mutant (*mus81-dd*). We updated S5 Fig. with the new data to maintain consistency of the genotypes across all assays with rrm3.

p. 9: The term “replication timing” is normally used to describe the temporal order of replication firing during S phase. Do the authors mean generation time?

Correction made.

p. 11, first line: Use Type I or Type II for consistency.

Update made.

p. 13. Fig S4 only shows inviability of cdc13-1 cells at 30 C, not G2-M arrest. Fig S4 could be removed. Numerous previous studies have documented the cdc13-1 growth defect at 30 C.

As requested, this figure has been omitted.

p. 22, line 11: non-permissive should be semi-permissive.

Correction made.

Reviewer #3: Schwartz et al. report that the structure-specific Mus81-Mms4 plays a role in the survival of yeast cells lacking telomerase, but not in either the Type I or Type II forms of repair. Hence the title strikes me as over-stated. I think most readers will interpret the claim that Mus81 is “required for telomere maintenance under replication stress” to mean that it is involved in telomere maintenance in the absence of telomerase or possibly even in the absence of telomere capping. Rather, the results seem to show that Mus81-Mms4 prevents accelerated senescence. To me that’s not the same thing and it makes the point that – despite the starting point of the authors, that Mus81 plays a role in mammalian ALT cells – Mus81 does not obviously play a result in yeast’s two “ALT” pathways. So my suggested title is” Mus81-Mms4 prevents accelerated senescence in telomerase-deficient cells.”

We agree with the reviewer and thank him/her for the title suggestion which we adopted.

In new data, the authors show some sort of synergy between agents that cause replication stalling (MMS and HU) and telomere dysfunction, as both est2 cells (well before senescence) and cdc13-1 cells show increased drug sensitivity in the absence of Mus81. Exactly what is being measured here is not clear. Both telomere damage and MMS/HU trigger a DNA damage response. It seems to me quite likely that the role of Mus81 in all of these assays is checkpoint-related and that combinations that increase the damage signal may impair the ability of haploid cells to “adapt” and to get to the point where survival through one of the repair pathways is possible. For example, Toczyski showed that cdc13-1 cells fail to resume cell division in mutants such as cdc5-ad or ckb1. Longhese showed similarly that overexpressing Ddc2, a key component of the DNA damage checkpoint, also prevented cells with a single chromosome break from resuming cell cycle progression. Since est2-deleted cells also elevate the checkpoint, perhaps the real problem here has nothing to do with recombination per se but with getting to the point where recombination can take place.

1. Hence it might be informative to repeat the assay done by Toczyski, using est2 and cdc13-1 in combination with cdc15-ts as a way to distinguish those cells that completed mitosis and those arrested at the G2/M boundary.

We appreciate the reviewer’s suggestion and have since updated our text to address this possibility.

p.22/23 - “What Mus81 is exactly doing under these conditions is unclear. Although there is supporting evidence that Mus81 is acting on replication intermediates during replicative stress, it is also possible that Mus81 is either contributing to or responding to checkpoint activation that happens under these conditions.”

2. The fact that mus81 est2 cells lose viability more rapidly can have two interpretations. First, there may indeed be some sort of accelerated inviability associated with replication problems near telomeres. Alternatively, there could simply be a delay in the formation of the first viable survivors.

We agree with the reviewer that our data is consistent with the possibility that Mus81 is contributing to the formation of the first viable survivors. Our submission mentioned this possibility in the discussion.

p. 20 line 18 - “Changes in the timing of cell density crisis in *est2Δ mus81Δ rad51Δ* and *est2Δ mus81Δ rad59Δ* triple mutants (Fig. 3B, C), suggests a partial involvement of *MUS81* telomerase-deficient survivor formation, but not an essential role as predicted.”

Does viability show the same decrease in a rad52 mutant, where there are no survivors and the drop in viable cells will measure the rate of a fatal telomere shortening?

The decrease in cell concentrations observed in *mus81 est2* is not nearly as severe as a *rad52 est2* deficient strain. The *rad52* mutant in the serial dilution senescence assay has an approximate 98% drop in cell viability after 24 hours (Meyer and Bailis, 2008), whereas *mus81 est2* mutants don’t start to show a reduced cell concentration relative to the *est2* single mutant until day 2 or 3.

By looking at telomere length directly, is there an obvious change in the distribution of telomere lengths at or before the time of “crisis”?

We have previously tried to do this experiment but were unable to overcome the challenge of DNA fragmentation and low cell densities at crisis. We never could get enough DNA to obtain clear Southern Blot results.

Fig. S3 looks at telomere length but since the gels are separate, I don’t think it’s possible to evaluate from these images whether there are more short telomeres in est2 mus81 than in est2 alone.

Supplemental Figure S3, panel E was data was to independently confirm that Mus81 was not required for yeast Type II survivors (p. 12 line 14). We do not make a comparison of the telomere lengths from this panel.

3. Mus81 is recruited to senescent telomeres. I agree it probably isn’t just a “passenger” but it is worth noting that Slx4 anchors a number of different nucleases and repair proteins and if it is recruited, its partners may come for the ride. Even inactive partners that still associate with the scaffold.

We agree that reference to the physical connection and potential recruitment between Slx4 and Mus81 is important for the interpretation of this study. Therefore, our original submission included 2 references to this interaction at the locations listed below. We include a full paragraph that discusses the recruitment possibility, highlighting that we see a role for both Mus81 and Slx1 at telomere, and that this role is unique to these two endonucleases, as both Yen1 and Rad1 do not display the same phenotype.

p. 24, line 12 – “In human cells, MUS81 accumulation at the telomere is dependent on the SLX4 endonuclease scaffold and telomere binding protein TRF2 [5, 6, 11]”

p. 4, line 19-22 – “In mammals, SLX4 physically interacts with a host of endonuclease complexes, including SLX1, MUS81, and XPF-ERCC1 (Rad1-Rad10 in budding yeast) [6, 9-13].”

p. 24, lines 11-23 – “Similar to human MUS81, *S. cerevisiae* Mus81 initially localizes to telomere DNA in the absence of functional telomerase (Fig. 6B). In human cells, MUS81 accumulation at the telomere is dependent on the SLX4 endonuclease scaffold and telomere binding protein TRF2 [5, 6, 11]. An interaction of Mus81-Mms4 with the Dpb11-Slx4 complex was reported, but this interaction is counteracted upon activation of the DNA damage checkpoint [92]. We were able to observe a role for Slx1 in cell growth and viability in the absence of functional telomerase, which was independent of *MUS81* (Fig. 1C; Fig. 4C; S2 Fig.). The involvement of Mus81 and Slx1 at the telomere was specific to these two structure-selective endonucleases and was not observed for either Yen1 or Rad1 – endonucleases that have been shown to cleave similar DNA structures *in vitro* and have overlapping roles in processing meiotic DNA structures. However, we cannot exclude the possibility that Yen1 or Rad1-Rad10 can partially compensate for the loss of Mus81 as has been seen in meiotic recombination [31]. Additional genetic analysis will need to be performed to evaluate overlapping roles for endonucleases in replication fork restart and telomere stability.”
