# Supplementary material for: Saccharomyces cerevisiae Mus81-Mms4 prevents accelerated senescence in telomerase-deficient cells
Source: PLoS Genet. 2020 May 29;16(5):e1008816. doi: 10.1371/journal.pgen.1008816 (PMC7286520; doi:10.1371/journal.pgen.1008816)
Supplement: Attachment — Submitted filename: Heyer mus81 PLoS review.docx [file pgen.1008816.s016.docx]

In this time of coronavirus, it is not reasonable to ask for some of the clarifying experiments that I and other reviewers suggested. So I will content myself to be picky about language. Three points, based on the authors’ responses.

1. It seems to me quite likely that the role of Mus81 in all of these assays is checkpointrelated

and that combinations that increase the damage signal may impair the ability of haploid

cells to “adapt” and to get to the point where survival through one of the repair pathways is

possible….

Hence it might be informative to repeat the assay done by Toczyski, using est2 and cdc13-1

in combination with cdc15-ts as a way to distinguish those cells that completed mitosis and

those arrested at the G2/M boundary.

*“What Mus81 is exactly doing under these conditions is unclear. Although there is*

*supporting evidence that Mus81 is acting on replication intermediates during replicative stress, it*

*is also possible that Mus81 is either contributing to or responding to checkpoint activation that*

*happens under these conditions.”*

***I suggest: Although there is supporting evidence that Mus81 is acting on replication intermediates during replicative stress, it is also possible that the absence of Mus81 enhances the DNA damage checkpoint and that cells may not be able to progress through the cell cycle and adapt to reach the point where they can form survivors (Xu et al. 2015, Coutelier et al. 2018)..”***

*Note that neither of these two important papers on telomerase deficiency and arrest have been cited*

2. Does viability show the same decrease in a rad52 mutant, where there are no survivors and the

drop in viable cells will measure the rate of a fatal telomere shortening?

*The decrease in cell concentrations observed in mus81 est2 is not nearly as severe as a rad52*

*est2 deficient strain. The rad52 mutant in the serial dilution senescence assay has an*

*approximate 98% drop in cell viability after 24 hours (Meyer and Bailis, 2008), whereas mus81*

*est2 mutants don’t start to show a reduced cell concentration relative to the est2 single mutant*

*until day 2 or 3.*

***Bailis’ results are outliers. See for example Chen et al. (2001). There is no explanation why rad52 would be different from rad51 rad59 double mutants – and in Chen et al there isn’t such a difference. This paragraph should be rewritten to note that several other labs have not observed the rad52 effect cited here. My original question was whether mus81 chenged the kinetics of inviability in rad52 (or rad51 rad59). Since the experiment hasn’t been done, perhaps the authors could simply consider if there would be such an effect.***

3. Mus81 is recruited to senescent telomeres. I agree it probably isn’t just a “passenger” but it is

worth noting that Slx4 anchors a number of different nucleases and repair proteins and if it is

recruited, its partners may come for the ride. Even inactive partners that still associate with the

scaffold.

*We agree that reference to the physical connection and potential recruitment between Slx4 and*

*Mus81 is important for the interpretation of this study. Therefore, our original submission*

*included 2 references to this interaction at the locations listed below. We include a full*

*paragraph that discusses the recruitment possibility, highlighting that we see a role for both*

*Mus81 and Slx1 at telomere, and that this role is unique to these two endonucleases, as both*

*Yen1 and Rad1 do not display the same phenotype.*

*p. 24, lines 11-23 – “Similar to human MUS81, S. cerevisiae Mus81 initially localizes to telomere*

*DNA in the absence of functional telomerase (Fig. 6B). In human cells, MUS81 accumulation at*

*the telomere is dependent on the SLX4 endonuclease scaffold and telomere binding protein*

*TRF2 [5, 6, 11]. An interaction of Mus81-Mms4 with the Dpb11-Slx4 complex was reported, but*

*this interaction is counteracted upon activation of the DNA damage checkpoint [92]. We were*

*able to observe a role for Slx1 in cell growth and viability in the absence of functional*

*telomerase, which was independent of MUS81 (Fig. 1C; Fig. 4C; S2 Fig.).*

***Again, the response doesn’t address my question. Slx4 is the scaffold. That slx1 has an effect different from mus81 doesn’t address the question whether slx4 deletion would have the same or a more profound effect (perhaps equivalent to mus81 slx1)***
